# Climate, weather, socio-economic and electricity usage data for the residential and commercial sectors in FL, U.S

**DOI:** 10.1016/j.dib.2017.05.031

**Published:** 2017-05-22

**Authors:** Sayanti Mukhopadhyay, Roshanak Nateghi

**Affiliations:** a Lyles School of Civil Engineering, Purdue University, West Lafayette, IN 47907, USA; b School of Industrial Engineering, Division of Environmental and Ecological Engineering, Purdue University, West Lafayette, IN 47907, USA

**Keywords:** Predictive energy analytics, Climate-energy nexus, Electricity consumption, Residential and commercial electricity sectors

## Abstract

This paper presents the data that is used in the article entitled “Climate sensitivity of end-use electricity consumption in the built environment: An application to the state of Florida, United States” (Mukhopadhyay and Nateghi, 2017) [1]. The data described in this paper pertains to the state of Florida (during the period of January 1990 to November 2015). It can be classified into four categories of (i) state-level electricity consumption data; (ii) climate data; (iii) weather data; and (iv) socio-economic data. While, electricity consumption data and climate data are obtained at monthly scale directly from the source, the weather data was initially obtained at daily-level, and then aggregated to monthly level for the purpose of analysis. The time scale of socio-economic data varies from monthly-level to yearly-level. This dataset can be used to analyze the influence of climate and weather on the electricity demand as described in Mukhopadhyay and Nateghi (2017) [1].

**Specifications Table**

**Table:** 

Subject area	*Energy*
More specific subject area	*Electricity demand, Climate change*
Type of data	*Table, Excel file*
How data was acquired	*Using different publicly available datasets such as: (i) U.S. Energy Information Administration (EIA) [form EIA-826]*[2]*; (ii) National Oceanic and Atmospheric Administration (NOAA); (iii) National Climatic Data Center (NCDC); (iv) U.S. Department of Labor; Bureau of Labor Statistics*[3]
Data format	*Raw; Aggregated, Filtered*
Experimental factors	*Not applicable*
Experimental features	*Statistical analysis of the data leveraging a range of parametric and non-parametric learning techniques to estimate the complex relationship between electricity demand and, climate non-stationarity and climate change*
Data source location	*Florida, United States*
Data accessibility	*Data is available within this article in the link provided*

**Value of the data**

• This dataset can be used for estimating the climate sensitivity of end-use electricity consumption in the residential and commercial sectors in FL, US.
• It can be also used to estimate the inadequacy risks in the electric power sector under climate variability and change.
• The aggregated and filtered data on end-use energy consumption, climate and weather variability, and socio-economic information can also be used by the scientific community to test various hypotheses of interest.
• It can also be used by researchers and data analysts who wish to leverage statistical or econometric modeling techniques to characterize climate-energy nexus in the residential and commercial sectors.

## 1 Data

The data presented in this article is included in a single excel file containing 35 variables. The excel file can be accessed from the link: https://engineering.purdue.edu/LASCI/research-data/energy. The variable measures are given in both Metric System of Measurement and Imperial System of Measurement. Table 1 summarizes the descriptions of all variables. This data contains valuable information related to electricity sales and revenue, electricity price, state-level climate and weather as well as socio-economic data obtained from four different data sources. The electricity sales data is trend-adjusted using the process as described in [1].

**Table 1:** Variable descriptions as contained in the dataset.

**Variable types**	**Variable names**	**Description**
**Electricity consumption**		
Electricity price	res.price	Electricity price in the residential sector (cents/kW h)
	com.price	Electricity price in the commercial sector (cents/kW h)
Electricity sales	res.sales.adj	Amount of electricity sales in residential sector trend-adjusted (GW h)
	com.sales.adj	Amount of electricity sales in commercial sector trend-adjusted (GW h)
**Climate variables**		
Degree days	HTDD	Heating degree days (Baseline=21.1 °C^a^)
	CLDD	Cooling degree days (Baseline=21.1 °C^a^)
Temperature	MMXT	Monthly mean maximum temperature (°C,°F)
	MNTM	Monthly mean temperature (°C,°F)
	MMNT	Monthly mean minimum temperature (°C,°F)
	EMXT	Extreme maximum daily temperature observed in a month (°C,°F)
	EMNT	Extreme minimum daily temperature observed in a month (°C,°F)
	DT90	Number days in a month with maximum temperature ≥32.2°C(90 °F)
	DT32	Number days in a month with minimum temperature ≤0°C(32 °F)
	DT00	Number days in a month with minimum temperature ≤−17.8°C(0 °F)
	DX32	Number days in a month with maximum temperature ≤0°C(32 °F)
Precipitation	EMXP	Extreme maximum daily precipitation observed in a month (mm, inches)
	TPCP	Total precipitation in a month (mm, inches)
	TSNW	Total snow fall in a month (mm, inches)
	MXSD	Maximum snow depth observed in a month (mm, inches)
	DP10	Number of days with ≥25.4 mm (1.0 in.) of precipitation
	DP01	Number of days with ≥2.54 mm (0.1 in.) of precipitation
	DP05	Number of days with ≥12.7 mm (0.5 in.) of precipitation
**Weather variables**		
Temperature	MDPT	Average monthly dew point temperature aggregated from daily dew point temperature observations (°C,°F)
Visibility	VISIB	Average daily meteorological visibility recorded over a month (km, miles)
Wind Speed	WDSP	Average daily wind speed recorded over a month (m/s, miles/hour)
	MXSPD	Average daily maximum sustained wind speed recorded over a month (m/s, miles/hour)
	GUST	Average daily wind gust recorded over a month (m/s, miles/hour)
**Socio-economic variables**		
Labor	LABOR	Labor force
Employment	EMP	Number of people in the labor force employed per month
	UNEMP	Number of people in the labor force unemployed per month
	UNEMPRATE	Unemployment rate per month (%)
	PCINCOME	Per capita income (USD)
	GSP	Real gross state product (million USD)

a The balance point summer temperature value depends on the state under investigation. It increases with decreasing latitude. Normally, the balance point temperature for states is 18.3^o^C (65^o^F) 65 °F. The only exception is Florida with the lowest latitude at the center of population, for which such a low base value is incapable of generating a good model. Florida presented an anomalous situation where appropriate balance point temperature was determined to be 21.1 °C (70 °F) [4].

## 2 Experimental design, materials and methods

The data on end-use energy consumption, climate and weather, and the socio-economic information were obtained from various publicly available data sources such as U.S. Energy Information Administration (EIA) [form EIA-826] [2], National Oceanic and Atmospheric Administration (NOAA), National Climatic Data Center (NCDC) and U.S. Department of Labor; Bureau of Labor Statistics [3] respectively. The data spans from 1990 to 2015. The monthly end-use electricity consumption data was trend-adjusted as described in [1]. The daily weather data obtained from various weather stations was first station-averaged and then aggregated to monthly level data. The socio-economic data included both monthly and yearly level variables. The variables such as labor, employment, unemployment and unemployment rate contain monthly-level information while the per capita income and the real gross state product are measured at yearly-levels. These yearly level variables are considered to be constant for all the months during that particular year. All the variables were then aggregated using the year and the months as the nexus.
