# Supplementary material for: Climate, weather, socio-economic and electricity usage data for the residential and commercial sectors in FL, U.S
Source: Data Brief. 2017 May 22;13:192–5. doi: 10.1016/j.dib.2017.05.031 (PMC5458065; doi:10.1016/j.dib.2017.05.031)
Supplement: Supplementary file 1 — Supplementary material [file mmc1.docx]

Conflict of Interest Form

No conflict
